# Use of extracorporeal hemoperfusion therapy in an adult horse with *Clostridioides difficile* colitis and severe systemic inflammatory response syndrome

**DOI:** 10.1111/jvim.17154

**Published:** 2024-08-09

**Authors:** Kallie J. Hobbs, Andre N. V. Le Sueur, Kimberly Hallowell, Emily Martin, Mary Katherine Sheats, Yu Ueda

**Affiliations:** ^1^ Department of Clinical Sciences College of Veterinary Medicine, North Carolina State University Raleigh North Carolina USA

**Keywords:** cytokines, equine, hemodialysis, hemoperfusion, immunology, renal/urinary tract

## Abstract

An 8‐year‐old American Quarter Horse gelding was treated with extracorporeal hemoperfusion (HP) therapy for treatment of *Clostridioides difficile* (*C. difficile*) colitis‐induced systemic inflammatory response syndrome (SIRS). The gelding developed *C. difficile* associated peracute colitis and severe SIRS as evidenced by a positive fecal *C. difficile* PCR and tachypnea, tachycardia, fever, neutropenia, altered mucous membrane color, and hyperlactatemia. Concurrent acute kidney injury in the horse limited the use of routine anti‐inflammatory and anti‐lipopolysaccharide treatments, including flunixin meglumine and polymyxin B, because of potential for nephrosis. Extracorporeal HP therapy was performed twice within 48 hours of the onset of severe SIRS during which the horse's physical examination variables stabilized. The horse was euthanized after 4 days because of laminitis. These findings support further investigation of extracorporeal HP therapy as an adjunctive treatment for severe SIRS/sepsis in horses.

**Abbreviations**
AKI: acute kidney injury
ECT: extracorporeal therapy
LPS: lipopolysaccharide
NSAID: non‐steroidal anti‐inflammatory drug
UHF: unfractionated heparin

## 1 INTRODUCTION

In horses, peracute colitis because of *Clostridioides difficile* (*C. difficile*) often leads to severe systemic inflammatory response syndrome (SIRS) and is associated with case fatality rates of 37%‐79%.^1^, ^2^, ^3^ Typical treatments for *C. difficile* colitis and secondary severe SIRS in horses include administration of non‐steroidal anti‐inflammatory drugs (NSAIDs), antimicrobials, anti‐lipopolysaccharide (LPS) therapy, and fluid therapy. In horses with renal compromise, these treatments are often limited because of the potential nephrotoxic effects of NSAIDs, aminoglycosides, and polymyxin B.

In human medicine, there is growing evidence that cytokines, including IFNγ, IL1‐β, and TNFα, act synergistically with *C. difficile* toxins A and B to damage enteric epithelium.^4^, ^5^ Severe elevations in cytokines (TNFα, IL‐10, IL1‐β) are associated with increased case fatality in horses and human ICU patients.^6^, ^7^ In humans with *C. difficile* colitis, elevated concentrations of TNFα are associated with a higher risk of death, and there is increasing evidence that cytokine targeting therapy could provide a novel pathway to decrease destruction of the intestinal epithelium by limiting cytokine and *C. difficile* interactions.^8^ One treatment method for reducing or eliminating cytokines is hemoperfusion (HP) therapy. Cytokine adsorption with HP was used frequently in human patients during the COVID‐19 pandemic to aid in managing cytokine storms initiated by sepsis.^6^ Extracorporeal cytokine adsorption therapy is described in small animals to treat heatstroke and immune‐mediated inflammatory conditions such as immune‐mediated hemolytic anemia.^9^, ^10^

This report describes the clinical use of extracorporeal HP therapy as an adjunct treatment for *C. difficile* colitis and severe SIRS/sepsis in a horse. Because of concurrent acute kidney injury (AKI), routine treatments such as flunixin meglumine and polymyxin B were contraindicated because of potential nephrotoxicosis, and novel treatment options were considered. Given evidence for cytokines as therapeutic targets for both *C. difficile* colitis and severe SIRS, the horse was identified as a candidate for HP therapy.

## 2 CASE DESCRIPTION

An 8‐year‐old, 670 kg, 17.1 hand high, BCS 5/9, American Quarter Horse gelding presented to the veterinary referral hospital for arthrodesis of the proximal interphalangeal joint for treatment of a medial palmar eminence fracture of the 2nd phalanx of the left forelimb that occurred 10 days before admission. Complete blood count and serum biochemistry panels were performed at admission and results were within normal limits. The horse was confirmed to be negative for HYPP, PSSM1, MH, GBED, and HERDA, according to AQHA documents. Therapy with phenylbutazone (2.2 mg/kg, IV, Q12) was initiated. Preoperatively, the horse was administered potassium penicillin (22 000 IU/kg, IV, Q6) and gentamicin (6.6 mg/kg, IV, Q24). During surgery, the horse experienced an acute episode of hyperkalemia (highest potassium concentration: 8 mmol/L) and hyperglycemia (highest glucose value: 350 mg/dL) that was treated with insulin (Vetsulin 40 U/mL, 0.01 unit/kg bolus), terbutaline (0.01 mg/kg, IM), and a 1 L bolus IV of 0.46% solution of calcium gluconate in Hartman's solution. The horse's serum potassium decreased to 3.8 mmol/L. The horse's serum creatinine concentration was (2.5 mg/dL, RI: 1.0‐1.7) 2 hours after recovery, an increase from 1.5 mg/dL on admission consistent with stage 1 AKI based on the VAKI staging system.^11^

Twenty‐four hours after‐surgery, the horse was comfortable on the operated limb but had an increased temperature 101.1°F./38.39°C and respiratory rate of 20 rpm. The horse became acutely tachycardic (66 bpm), and tachypneic (40 rpm), with brick red injected mucous membranes, full body muscle fasciculations, and further elevation in body temperature to 103.3°F (39.6°C). Ultrasonographic examination revelated fluid throughout all visible portions of the large colon. Complete blood cell count revealed hemoconcentration, marked leukopenia, and marked neutropenia. Coagulation variables were mildly prolonged. Biochemistry revealed an elevated creatine kinase activity, normal total protein, and persistently elevated serum creatinine concentrations (Table 1). Fecal PCR testing was positive for *C. difficile* toxin B. Fecal Salmonella culture and PCR were negative. Other fecal testing diagnostics were not performed because of financial limitations.

**TABLE 1:** Selected complete blood cell count and biochemistry values from Day 1, Day 2, and Day 3 of hospitalization.

Day	Packed cell volume (%)	White blood cell count: cells/μL	Neutrophil count: cells/μL	Prothrombin time: seconds	Activated partial thromboplastin time: seconds	Lactate: mmol/L	Creatine kinase: IU/L	Creatinine: mg/dL	Total protein: g/dL
Day 1	53	1.88 × 10^3^	0.289 × 10^3^	13.3	55.9	6.4	3489	2.6	6.0
Day 2	43	2.88 × 10^3^	1.073 × 10^3^	X	X	2.2	1189	2.6	4.6
Day 3	41	4.6 × 10^3^	2.6 × 10^3^	X	X	X	382	1.9	3.7

*Note*: Reference intervals are as follows: Packed cell volume: 28%‐46%, White blood cell count: 4.69‐10.36 × 10 cells/μL, neutrophil count: 2.4‐6.8 × 10 cells/μL, prothrombin time: 10.3‐12.9 seconds, activated partial thromboplastin time: 37.3‐51.4 seconds, creatine kinase: RI: 117‐564 IU/L, and total protein: 5.9‐8.0 g/dL.

With a working diagnosis of *C. difficile* peracute colitis, severe SIRS/sepsis and AKI, supportive care was initiated, including parenteral fluids which included a 10 L bolus of isotonic fluids (IV Hartmann's Solution, Dechra, Northwich, England) and a 1 L bolus of fresh frozen equine plasma (IV Protein Volume Replacer, MG Biologics, Ames, Iowa) followed by a CRI rate of 1‐3 L/hour of isotonic fluids, which was adjusted to attain, and then maintain hemodynamic stability. Additional plasma was not administered because of owner limitations. Clopidogrel was administered for prevention of thrombophlebitis^12^ (4 mg/kg as a loading dose and followed by 2 mg/kg, PO Q24). Potassium penicillin treatment was continued, and gentamicin treatment was replaced with enrofloxacin (5.5 mg/kg, IV Q24) because of concern for continued administration of aminoglycosides in the face of AKI. The horse was maintained on a CRI of ketamine (0.5 mg/kg/hour) and lidocaine (0.05 mg/kg/minute) for post operative pain. Novel treatments to address SIRS were considered, specifically HP therapy. Owners were informed that preliminary studies show that HP was safe and decreased LPS‐induced equine cytokines ex vivo, but that no clinical data on HP efficacy in horses was available.^13^, ^14^ The owners elected for the horse to receive extracorporeal HP therapy.

The horse had a 14 Fr × 25‐cm double‐lumen temporary hemodialysis catheter (Med‐Comp Silicone Hemo‐Cath) placed sterilely in an external right jugular vein without complication using the modified Seldinger technique under no sedation. The HP sessions were performed on a VetSmart machine (Medica‐Spa, Medolla, Italy), and the blood purification column chosen was a polymer‐based VetResQ, Cytokine, and Lipophilic Drug Removal—300 mL device (CytoSorbents Inc, Monmouth Junction, New Jersey).

Systemic anticoagulation was achieved with a starting dose of 50 IU/kg/IV dose of unfractionated heparin (UFH). Activated clotting time (ACT) was measured using a Medtronic ACT 2 machine and maintained within the range of 500‐900 seconds. The ACT range was determined by targeting a 2.5× from baseline time, as is common in other species undergoing extracorporeal therapies,^15^ and incorporating clinician experience with HP in horses. The horse's baseline ACT was 250 seconds (RI: not reported).

In the 1st session, the total blood volume processed was 35.4 L (66% estimated blood volume) (blood flow rate range of 80‐200 mL/minute). The blood flow rate was adjusted to ensure arterial and venous chamber pressures were maintained in the range of −250 to +200 mm Hg. The horse received supplemental UFH via CRI (20 IU/Kg/h/IV) throughout treatment to support appropriate anticoagulation within the extracorporeal circuit. The HP column was replaced every 120 minutes to avoid saturation, as the maximum filtration/binding capacity of the column has not been determined in the horse.

During the 2nd hour of the HP therapy, observations included a moderate improvement in physical examination (Temp: 101.5 F [38.61°C], Resp: 32 rpm, HR: 60 bpm, MM: bright pink) and increased appetite for hay. At the end of the session (240 minutes), the horse's body temperature was 101.2 F, plasma lactate concentration decreased to 2.2 mmol/L, and the horse appeared brighter. The horse received 2 L of plasma IV throughout the 1st column of treatment in order to combat albumin loss from HP as well as protein‐losing enteropathy. Additional plasma was not given because of owner‐initiated financial restrictions.

The following day, CBC and biochemistry variables showed moderate improvement (Table 1). Because of ongoing signs of SIRS (leukopenia, mildly injected mucous membranes, tachycardia) (60 bpm, hyperthermia (101.7 F [38.7°C])), a 2nd session of HP was initiated 18 hours after the 1st treatment. Because of limited column availability, the 2nd session lasted 300 minutes, and HP columns were changed every 150 minutes rather than every 120 minutes, and the total blood volume processed in the 2nd session was 52.2 L (97.4% blood volume). After the 2nd treatment, the horse was bright with normal appetite, temperature (99.8 F [37.6°C]), and respiratory rate (20 rpm). Tachycardia (60 bpm) and altered mucous membrane color (bright pink) remained.

On the 3rd day after‐surgery, CBC and biochemistry variables were within reference intervals, except for hypoproteinemia (Table 1). Fecal consistency had improved from a watery to fibrous consistency, and elimination frequency had returned to normal. Intravenous fluid therapy, including the ketamine CRI, was discontinued. Because of the horses' improved systemic status, it was determined that a 3rd HP session was not clinically indicated.

**FIGURE 1:**
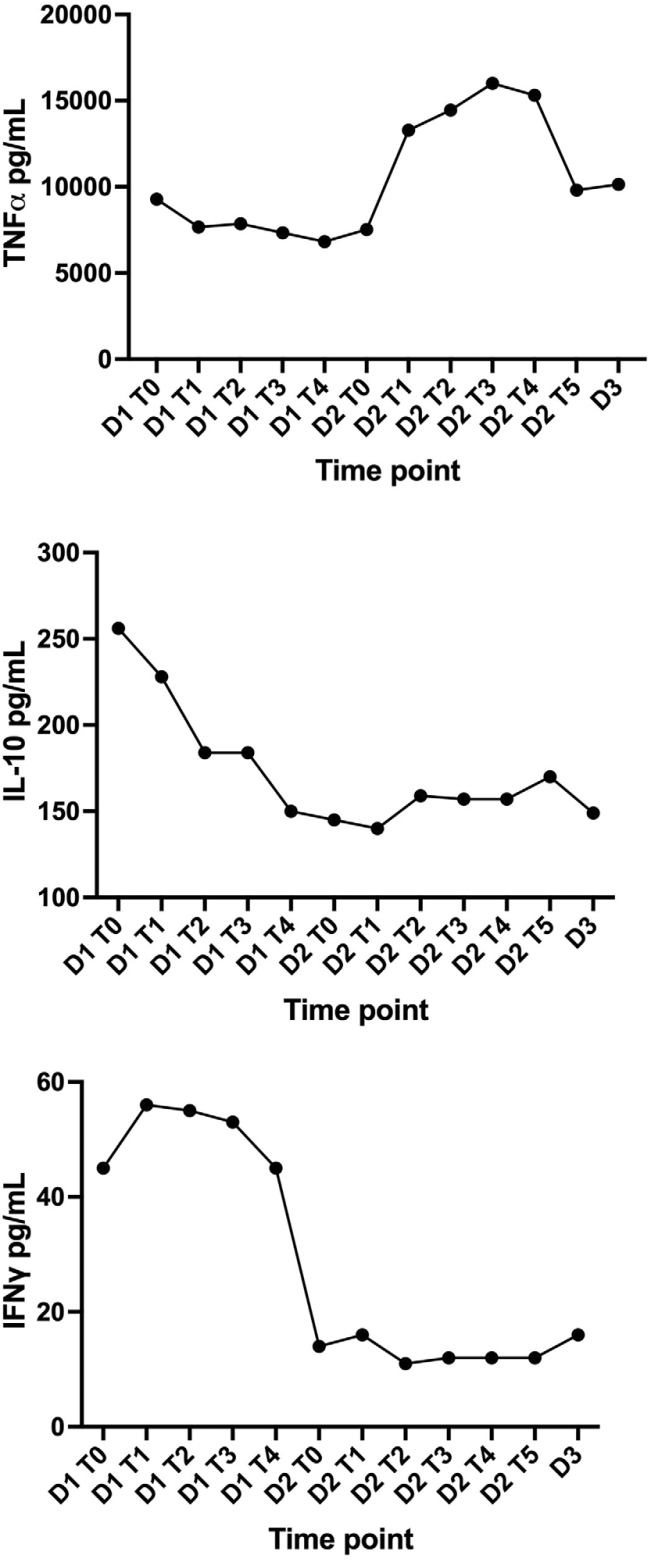
Concentrations of TNFα, IL‐10, and IFNγ over a 3‐day period. (D1) designates the 1st day hemoperfusion session. (D2) designates the 2nd day hemoperfusion session and (D3) designates Day 3 cytokine concentrations (no hemoperfusion performed). T designates time in hours.

During the 1st and 2nd HP treatments, blood samples were collected pre‐circuit at 0, 60, 120, 180, and 240 minutes. On Day 2, the baseline sample was collected several hours before initiation of treatment. Blood samples were immediately centrifuged at 1200×*g* for 10 minutes, and serum was harvested and frozen at −80°C. Within approximately 2 weeks, samples were analyzed for TNFα, IL‐1β, IL‐10, and IFNγ by equine cytokine and chemokine multiplex (Cornell University) (Figure 1). There were no measurable results for IL‐1β (data not shown).

The horse was hospitalized for a total of 4 days after the development of severe SIRS/sepsis. The horse remained comfortable on the injured limb and was fully weight‐bearing during this time. On the evening of Day 3, the horse was noted to exhibit forelimb weight shifting, which progressed to weight shifting on all 4 limbs by the morning of Day 4. Radiographs of all 4 ft and a venogram^16^ of the right front (contralateral) foot showed that P3 was normally positioned within all feet. However, there was reduced blood flow to the toe and dorsal surface of P3 in the right forelimb, consistent with acute laminitis. As the horse's future for athletic performance could not be guaranteed, the owners elected for euthanasia. A PCR for *C. difficile* toxin was repeated at the time of euthanasia and was negative.

## 3 DISCUSSION

This report describes the novel use of HP as an adjunctive treatment for an adult horse with *C. difficile* associated colitis and severe SIRS. Hemoperfusion is a novel treatment in the horse, but was specifically offered for this horse because of AKI and concern for potential nephrotoxicity of standard‐of‐care therapies.

Therapeutic attenuation of the cytokine response using HP has been investigated in human sepsis.^6^ The key mechanism behind this therapy is the restoration of cytokine balance, which then helps promote appropriate immune responses.^15^ For *C. difficile* colitis specifically, cytokines play an important role in the pathophysiology in both humans and mice.^17^, ^18^, ^19^ Therefore, it is possible that HP therapy and reduction of cytokine concentrations in horses with *C. difficile* colitis could benefit horses by reducing ongoing intestinal injury and inflammation and decreasing *C. difficile* disease severity.

In the case reported here, on Day 1, there was a 26.4% reduction in TNFα, a 41.4% decrease in IL‐10, and a 0% change in IFNγ. On Day 2, there was minimal to no reduction in measured cytokines. While HP could have contributed to these changes, the decreases seen on Day 1 could also have been because of normal elimination or anti‐inflammatory responses. The lack of change on Day 2 could result from a return to stable or baseline production, an increased rate of elimination to match production (potentially including a contribution of HP), or a lack of adequate anti‐inflammatory signals. This could be a product of the rebound effect of cytokines, which results from an imbalance of inflammatory and anti‐inflammatory cytokines, though this has not been reported with the use of VetResQ technology. Interestingly, TNFα increased before decreasing by the end of the treatment period. The reason for this pattern is unclear but could be because of increased TNFα production in the horse, oversaturation of the column, or both. In humans, the saturation time is typically 12‐24 hours, but there is a much smaller blood volume processed during this time in human patients. In this equine patient, filters were changed every 120‐150 minutes because of the limited number of available filters. In prior research with LPS induction models, systemic cytokine concentrations typically show a cytokine‐dependent increase over the initial 1‐12 hour period.^20^ As the baseline sample on Day 2 was collected as part of the horse's morning bloodwork, and therapy was not started until several hours later, this could explain the higher concentrations of cytokines present in the 1st hour of filtration.

Typically, a prescription is given for ECT; however, prescriptions are not currently standardized for HP.^21^ In this report, maximum filtration volume was limited by the use of pediatric lines (max flow 200 mL/sec), which were the only lines available at the time of treatment. Additionally, removal kinetics of cytokines in the horse have not been determined; therefore, prescriptions as used in other ECT modalities cannot be calculated. A recent study demonstrates that VetResQ removes cytokines from equine plasma ex vivo, with the highest removal in the 1st hour.^14^ However, ex vivo and in vivo removal kinetics might not be comparable. The difference between blood volume processed on Day 1 vs Day 2 was attributed to the horse's hypercoagulable state on Day 1, leading to increased clotting and a decreased absorption rate.

The column used in this horse contains porous polymer beads that capture cytokines and other molecules, such as clostridial toxins and Shiga toxin, by size exclusion and hydrophobic interactions in a concentration‐dependent and saturable process.^22^ Besides cytokines, it is also possible that HP removed bacteria or bacterial toxins from this horse's systemic circulation. While complete blood cell count and biochemistry variables in the horse improved over the course of treatment, with the exception of total protein. The improvement of CBC variables might be linked to decreased concentrations of cytokines such as TNFα, which results in the demargination of neutrophils.^23^ Other mechanisms that could have contributed to increasing neutrophil counts include increased bone marrow production or delayed apoptosis, which are both known to affect neutrophil numbers during sepsis.^24^

## CONFLICT OF INTEREST DECLARATION

Our group has an ongoing study using VETRESQ cartridges in large animals (funded through American Association of Equine Practitioners). The cartridges were donated with no intention or promise of publication from us. The Thoroughbred Education and Research Foundation donated money to help with clinical cases, they had no intention or promise of publication from the authors.

## OFF‐LABEL ANTIMICROBIAL DECLARATION

Authors declare no off‐label use of antimicrobials.

## INSTITUTIONAL ANIMAL CARE AND USE COMMITTEE (IACUC) OR OTHER APPROVAL DECLARATION

Authors declare no IACUC or other approval was needed.

## HUMAN ETHICS APPROVAL DECLARATION

Authors declare human ethics approval was not needed for this study.
